# Challenging the standard model by high-precision comparisons of the fundamental properties of protons and antiprotons

**DOI:** 10.1098/rsta.2017.0275

**Published:** 2018-02-19

**Authors:** S. Ulmer, A. Mooser, H. Nagahama, S. Sellner, C. Smorra

**Affiliations:** Ulmer Fundamental Symmetries Laboratory, RIKEN, Wako, Saitama 351-0198, Japan

**Keywords:** antiproton, proton, charge–parity–time tests, Penning traps

## Abstract

The BASE collaboration investigates the fundamental properties of protons and antiprotons, such as charge-to-mass ratios and magnetic moments, using advanced cryogenic Penning trap systems. In recent years, we performed the most precise measurement of the magnetic moments of both the proton and the antiproton, and conducted the most precise comparison of the proton-to-antiproton charge-to-mass ratio. In addition, we have set the most stringent constraint on directly measured antiproton lifetime, based on a unique reservoir trap technique. Our matter/antimatter comparison experiments provide stringent tests of the fundamental charge–parity–time invariance, which is one of the fundamental symmetries of the standard model of particle physics. This article reviews the recent achievements of BASE and gives an outlook to our physics programme in the ELENA era.

This article is part of the Theo Murphy meeting issue ‘Antiproton physics in the ELENA era’.

## 1. Introduction

As a consequence of the combined charge, parity and time reversal (CPT) invariance [1] of the Lorentz-invariant local quantum field theories of the standard model, particles and their antimatter counterparts have identical masses, lifetimes, charges and magnetic moments, the latter two of opposite sign. Comparisons of the fundamental properties of matter/antimatter conjugates with high precision constitute a challenge to the standard model, because any measured difference would hint at new physics. This is the motivation and inspiration to perform different direct CPT tests of this kind. Decay channels of the neutral 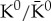 mesons have been compared [2], which constrained the relative mass difference of the particles to 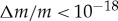. Penning-trap-based single-particle experiments compared the electron and positron (*g* − 2) values with a precision better than four parts per billion (ppb) [3], which allowed their *g*-factors to be constrained with a fractional precision of about two parts per trillion (ppt). In the lepton sector, another precise test of CPT invariance was carried out: the *g* − 2 values of μ^+^ and μ^−^ have been found to be identical at a level of 4 ppb [4]. Interestingly, the measured muon *g*-factors deviate by 3.6 standard deviations from the standard model prediction.

The experiments located at the Antiproton Decelerator (AD) of CERN contribute to this endeavour. The ALPHA [5], the ATRAP [6] and the ASACUSA [7] collaborations target 1S/2S and ground-state hyperfine spectroscopy of the simplest atom that is entirely made out of antimatter—antihydrogen. Very recent dramatic progress in trapping of antihydrogen led to exciting results. The ALPHA collaboration reported on the first detection of optical transitions in antihydrogen [5], and presented a ground-state hyperfine-splitting measurement at the level of 350 parts per million (ppm) [8]. ASACUSA reported on the production of a beam of antihydrogen atoms [7] which is a major step towards a ground-state hyperfine-splitting measurement in a beam. A second experiment in ASACUSA is dealing with laser spectroscopy of antiprotonic helium, giving access to the antiproton-to-electron mass ratio, which was recently determined with an uncertainty of 0.8 ppb [9].

Another branch of experiments, a part of ATRAP and BASE, investigates the fundamental properties—lifetime, charge-to-mass ratio and magnetic moment—of single trapped protons and antiprotons stored in Penning traps. In 1999, as a final result of a pioneering, years-long experiment campaign, the precursor experiment of ATRAP reported on a proton-to-antiproton charge-to-mass ratio comparison with a fractional precision of 90 ppt [10], and in 2012, ATRAP measured the antiproton magnetic moment with a fractional resolution of about 5 ppm [11].

BASE [12] was approved in 2013, and in 2014 performed a proton-to-antiproton charge-to-mass ratio comparison at a fractional precision of 69 ppt [13] and, at our companion experiment at Mainz, Germany, the most precise measurement of the magnetic moment of the proton with a fractional precision of 3 ppb [14]. In 2015/2016, we have invented [15] and successfully implemented [16] a reservoir trap technique for antiprotons, demonstrated trapping of antiprotons for 405 days, and derived from this measurement the most stringent limit on directly measured antiproton lifetime [17]. In 2017, we reported on the most precise measurement of the magnetic moment of the antiproton with a fractional precision of 0.8 ppm [18], and detected the first single antiproton spin transitions [19], which is a major step towards an at least 100-fold improved measurement of the antiproton magnetic moment.

This article summarizes the results produced by BASE since 2013, when the project was approved by CERN's research board, and gives an outlook on our experiment programme in the ELENA era.

## 2. Materials and methods

### (a) Penning trap

BASE uses an advanced Penning trap system to investigate and compare the fundamental properties of single trapped protons and antiprotons. In a Penning trap, a homogeneous magnetic field *B* in the axial direction (*z*) is superimposed with an electrostatic quadrupole potential 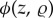, 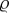 being the radial coordinate. In such a configuration of static fields, a charged particle describes a trajectory which is a superposition of three independent harmonic oscillator motions. The modified cyclotron motion at frequency 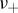 and the magnetron motion at frequency 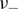 are perpendicular to the magnetic field axis, and the axial motion at frequency 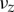 oscillates along the magnetic field lines. An invariance theorem 
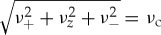
 relates the trap eigenfrequencies to the free cyclotron frequency 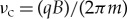, which is defined by the fundamental properties, charge *q* and mass *m*, of the trapped particle [20]. If the particle carries spin, the associated magnetic moment precesses in the magnetic field of the trap with the Larmor frequency 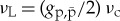, where 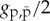 is the magnetic moment 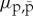 of the trapped proton/antiproton in units of the nuclear magneton 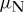.

For protons p and antiprotons 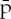, the Penning trap therefore provides access to two types of measurements:

(i) charge-to-mass ratio comparisons, by measuring the cyclotron frequencies 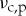 and 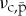,

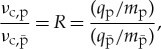

and

(ii) determinations of magnetic moments 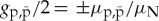 by measuring 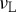 and 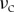.

The determination of 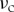 is based on image current detection, which is nowadays a straight-forward standard method relying on highly sensitive superconducting tuned circuits [21], while the measurement of the Larmor frequency 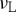 relies on the technically challenging application of the continuous Stern–Gerlach effect [3].

### (b) Continuous Stern–Gerlach effect

**Figure 1.**
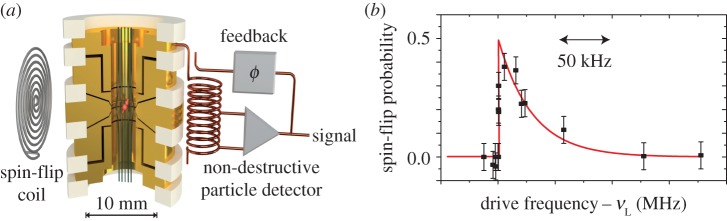
(*a*) Penning trap with a superimposed magnetic bottle for the detection of proton and antiproton spin quantum transitions. (*b*) Larmor resonance as measured in [18]. The steep slope represents the Larmor or spin precession frequency. (Online version in colour.)

Unlike in cyclotron frequency measurements, the Larmor precession of the particle's spin is not accompanied by a shift of charge. Therefore, it cannot be detected by direct image current methods. To access the Larmor precession, we superimpose a magnetic bottle 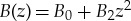 on the Penning trap (figure 1*a*). This adds a spin-dependent magnetic axial potential to the axial electrostatic trapping potential. Accordingly, the axial oscillation frequency becomes a function of the spin eigenstate,

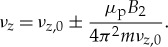

This method has been applied with great success in measurements of electron/positron magnetic moments [3]. The difficulty in applying these methods to the proton/antiproton is related to the 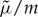 scaling of the axial frequency jump 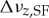 [22]. For protons and antiprotons, this ratio 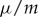 is about 10^6^ times smaller than for the electron/positron. To tackle this challenge, we impose on our trap a magnetic bottle with a strength of 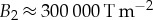. In this extreme magnetic environment, a proton/antiproton spin transition induces an axial frequency shift of only 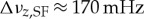 out of typically 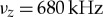.

Once an apparatus has been designed which enables the detection of the axial frequency shifts induced by spin transitions, the spin-flip probability as a function of an applied radio-frequency (rf) can be measured [22]. The result leads to a well-understood line-shape [23], as shown in figure 1*b*, from which 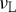 is extracted. The same principle can be applied to measure the cyclotron frequency 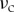 [18].

The development of the methods which enable the detection of these tiny axial frequency shifts in the presence of the ultra-strong 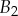 required several years of dedicated research and development work and is documented in a sequence of publications in which we have demonstrated the first observation of spin flips with a single trapped proton [22] as well as the first high-fidelity detection of single-proton spin transitions [24]. Related work by another effort has been published in [11].

### (c) Multi-trap methods

#### (i) Magnetic moment measurement in a double trap

**Figure 2.**
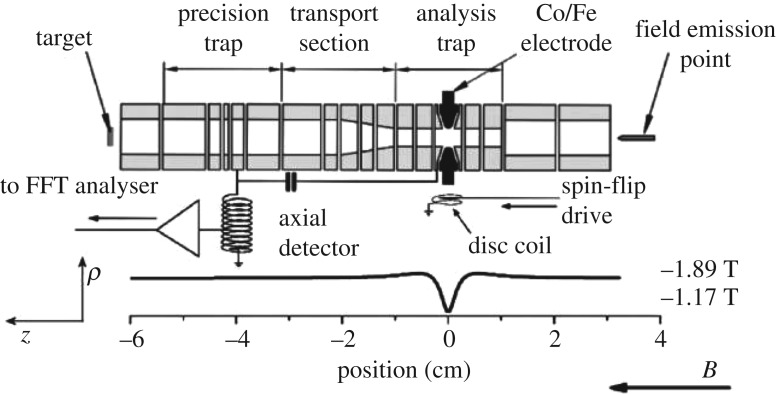
Double Penning trap as used in the BASE experiment at the University of Mainz, Germany [14,22]. The trap assembly consists of a homogeneous precision trap for frequency measurements and an inhomogeneous analysis trap, with a superimposed magnetic bottle for proton spin state analysis. A detailed description of a double-trap measurement sequence is described in the text.

The above-explained principle to determine the *g*-factor of a single proton/antiproton in a Penning trap with a superimposed magnetic bottle is limited by the bottle itself, which produces a considerable line-width. Quantum transitions in the magnetron oscillator mode limit the determination of 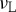 and 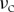 to the ppm level [18]. To overcome this limitation, Häffner *et al*. [25] introduced the double Penning trap technique. This method has been developed to enhance measurement precision in determinations of the *g*-factor of the electron bound to highly charged ions. BASE adapted this method and successfully developed a double Penning trap instrument [14] with 30-fold improved sensitivity with respect to magnetic moments (figure 2) [22].

In the double-trap method, a homogeneous precision Penning trap (PT) is added to the trap assembly. In this PT, the magnetic field is about 100 000 times more homogeneous than in the trap with the superimposed magnetic bottle (analysis trap (AT)). In a double-trap measurement sequence, the spin state is identified in the AT; afterwards the particle is transported to the precision trap, where its cyclotron frequency 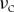 is measured while spin transitions are induced by applying a magnetic rf drive at 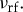 Subsequently, the particle is transported back to the AT where its spin state is analysed. By repeating this sequence many times at different drive frequencies, the spin-flip probability as a function of 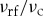 is obtained. Compared to single-trap measurements, the homogeneous magnetic field in the PT drastically narrows the width of the *g*-factor resonance, typically enabling measurements on the ppb level [14]. The application of the double-trap method requires high-fidelity spin state detection, which means that the spin state of the particle can be clearly determined within two axial frequency measurements, constituting the major challenge in such measurements [24].

#### (ii) Charge-to-mass ratio measurement in a triple trap

**Figure 3.**
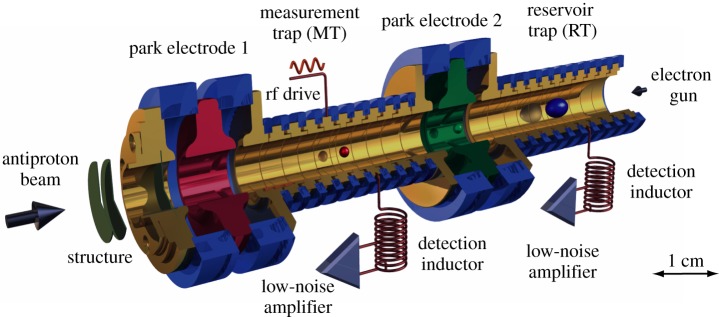
Multi-trap assembly as used in the proton-to-antiproton charge-to-mass ratio comparison, the first high-precision Penning trap experiment in which the ‘fast shuttling’ method was applied [13]. The image also shows the reservoir trap, which enables antiproton experiments, independent of accelerator up-times.

Classical mass spectrometers usually use single measurement traps. In frequency-ratio measurements, the particles are loaded from external or *in situ* sources; this re-loading/preparation procedure typically takes several tens of minutes. An elegant update to this classical method has been introduced by Gabrielse *et al.*, which compared cyclotron frequencies of antiprotons and negatively charged hydrogen ions [10]. In this experiment, both particles were stored in the same trap, the measurement particle in the centre and the second particle on a large orbit. Including particle swapping, a single frequency-ratio measurement typically required 4 h. In [13], we have applied a novel three-trap method, which uses two park traps and one measurement trap, as illustrated in figure 3.

Here we prepare a particle in the centre of the measurement trap and another one in a park electrode. Shuttling of this particle configuration along the magnetic field axis enables particle exchange within typically 10 s. By combining this fast shuttling technique with sideband measurement methods to determine the cyclotron frequency, we were able to perform one single frequency-ratio measurement in 240 s, which corresponds to a 60-fold improved ratio sampling rate, compared to previous proton/antiproton charge-to-mass ratio comparisons.

## 3. Physics

### (a) Reservoir trap and antiproton lifetime

**Figure 4.**
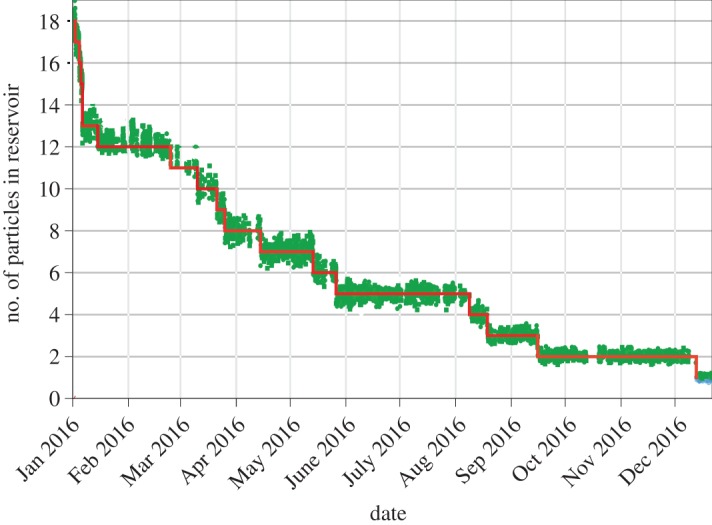
Results of continuous monitoring of the BASE reservoir trap content. The steps correspond to controlled extractions to supply antiprotons to the adjacent high-precision measurement traps. In the entire 2016 run, we consumed in total 18 antiprotons. In summary, in the 2015/2016 BASE antiproton run, trapping of antiprotons was demonstrated for 405 days. (Online version in colour.)

BASE implemented the unique reservoir trap (RT) method [15,16]. In this trap, a cloud of antiprotons is stored, which allows us to perform antiproton experiments independently from accelerator up-times. This device even allows antiproton experiments during CERN's annual shut-down, when the background magnetic field fluctuations in the AD hall are low. The reservoir trap also provides the possibility to constrain directly measured antiproton lifetime. In 2015/2016, we demonstrated antiproton trapping for about 405 days. Figure 4 shows the content of the reservoir trap as a function of time; steps are related to documented extractions. None of the steps is caused by an antiproton decay or annihilation with background gas. By combining all available reservoir trap datasets, we extract a lower antiproton lifetime limit of 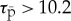 a [17]. Compared to other CPT tests, antiproton lifetime measurements test the conservation of the baryon number. In 1999, the APEX collaboration set limits on several antiproton decay channels in a storage ring [26]. Cosmic ray studies have set antiproton lifetime constraints based on model-dependent antiproton production mechanisms in the cosmos [27]. By contrast, our measurement sets the best directly measured limits on the full half-life of the antiproton. It is sensitive to potentially invisible decay channels which are favoured by some grand unified theories [28,29]. BASE plans to further push the limit in 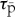 by improving the trapping rate by a factor of 10–100.

### (b) Proton-to-antiproton charge-to-mass ratio

With the fast shuttling technique which has been described above, we performed in the 2014 antiproton run about 6500 cyclotron-frequency-ratio comparisons of antiprotons and negatively charged hydrogen ions [13]. Using hydrogen ions instead of protons drastically reduces systematics in the frequency-ratio comparisons [10,13]. The hydrogen ion serves down to the sub-ppt level as a perfect, negatively charged, proxy of the proton, its mass being

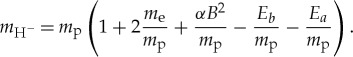

Here the first correction term is the electron-to-proton mass ratio, the second a polarizability shift; the last two corrections account for the binding energies of the electrons. This leads to a theoretically expected cyclotron frequency ratio 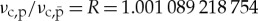. From our measurements, we extract

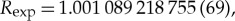

which is, within the achieved experimental uncertainty, consistent with CPT invariance:

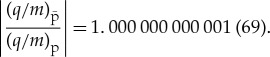

**Figure 5.**
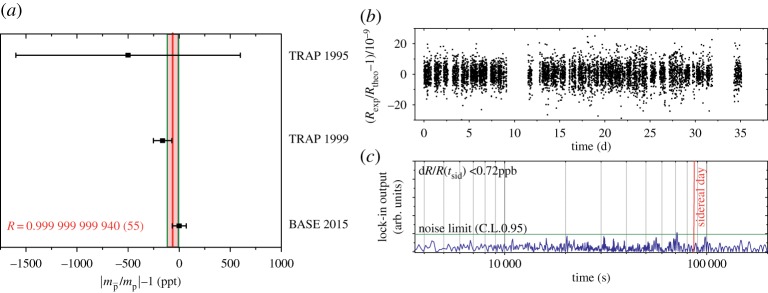
(*a*) Measurements of the antiproton-to-proton charge-to-mass ratio. The 1995 measurement (ppb) by the TRAP collaboration compared proton and antiproton cyclotron frequencies. The 1999 TRAP measurement (90 ppt) and the 2014 BASE measurement (69 ppt) compared antiproton and H^−^ cyclotron frequencies. (*b*) Measured frequency ratios and (*c*) output of lock-in analysis applied to the data shown in (*b*).

Combining all the available comparisons of the proton-to-antiproton charge-to-mass ratios (figure 5*a*) leads to an unconstrained weighted average of

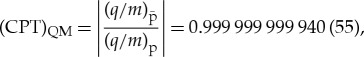

which is within 1.2 standard errors (68% confidence level) consistent with CPT invariance. The high sampling rate in our charge-to-mass ratio measurements also enables the search for sidereal variations. Such variations are possible by Lorentz-violating extensions of the standard model. By performing a lock-in analysis to the dataset shown in figure 5*b*, we obtain the output shown in figure 5*c*, which sets to any Lorentz-violating diurnal effect a limit 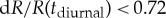 ppb.

Given the assumption that CPT invariance holds, the above measurement allows for a test of the weak equivalence principle (WEP). In this case, the proton/antiproton cyclotron frequencies are, in the absence of a gravitational field, identical by definition. If a gravitational field is being introduced while the WEP is broken to some extent, the proton and the antiproton will experience a different gravitational red-shift. The combined value 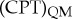 constrains 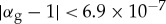, the parameter 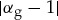 representing a possible gravitational anomaly acting on antimatter [30]. Note that we follow here the previously published literature, which uses the gravitational potential of the local galactic supercluster, an assumption which has been controversially discussed. To set direct constraints on the gravitational red-shift, which do not require additional assumptions, e.g. by performing charge-to-mass ratio measurements at different altitude, a fractional resolution on the 0.01 ppt scale would be required. The AEgIS, ALPHA-g and GBAR experiments at CERN are planning to investigate the WEP using antihydrogen. Their expected results will provide important information to disentangle the limits on WEP and CPT violation from proton/antiproton cyclotron frequency comparisons.

### (c) Proton and antiproton magnetic moments

Several years of research and development work were required to implement the methods to compare the magnetic moments of the proton and the antiproton. In this context, we have reported on the first observation of spin flips with a single trapped proton [22], the first observation of single spin transitions of a single proton [24] and the first successful demonstration of the double Penning trap technique [31]. These achievements culminated in the first direct high-precision measurement of the proton magnetic moment [14] in units of the nuclear magneton. From the recorded double-trap *g*-factor resonance, we obtain

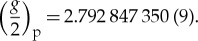

This result constitutes the most precise measurement of the proton magnetic moment to date, agrees with the previous best value which was extracted from hydrogen maser spectroscopy [32], but is about three times more precise.

One of the major goals of the BASE collaboration is the application of the double Penning trap technique to measure the magnetic moment of the antiproton at a comparable level of precision. The implementation of this measurement in the noisy environment of an accelerator hall is a highly challenging task. In a first step towards this major goal, we have performed a single Penning trap measurement of the antiproton magnetic moment, relying on frequency measurements in the strong magnetic bottle. In that measurement campaign, we have recorded in total 12 cyclotron resonances, interleaved by Larmor resonance scans [18]. The resulting *g*-factor which was obtained from this measurement campaign,

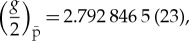

has a fractional precision of 0.8 ppm at 95% confidence level. This measurement improves the fractional precision of the previous best value [11] by a factor of six, and is consistent with CPT invariance:

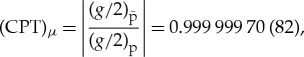

limited by the measurement precision of the antiproton magnetic moment.

**Figure 6.**
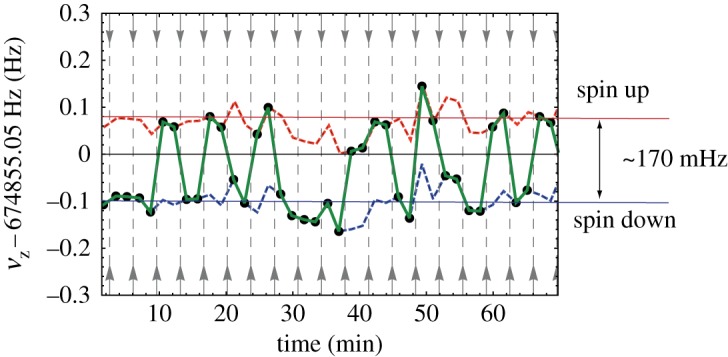
First observation of single spin flips with a single trapped antiproton. The axial frequency is measured sequentially; after each axial frequency measurement, a spin-flip drive is applied. The spin up and the spin down states can be clearly distinguished. Given the current experimental conditions, the spin state detection fidelity is at 91.2%, which is sufficient to apply the double Penning trap method. (Online version in colour.)

With the successful application of the double Penning trap technique to measure the magnetic moment of the antiproton, an at least 100-fold improved CPT test with proton/antiproton magnetic moments will become possible. Very recently, a major step towards this challenging goal has been achieved. For the first time, we have observed single antiproton spin transitions [19]. A representative result on the unambiguous detection of such antiproton spin flips is shown in figure 6.

In the experiment, the axial frequency of a single antiproton is measured in the magnetic bottle of the analysis trap. After each frequency measurement, a resonant rf drive is applied which induces spin flips. The results shown in figure 6 clearly demonstrate the first non-destructive observation of quantum transitions in baryonic antimatter, and are the most important prerequisite for the application of multi-Penning trap methods to measure the antiproton magnetic moment.

## 4. Discussion

Several models exist which provide a framework to interpret and compare sensitivities of tests of CPT invariance [33], the most prominent of which is the standard model extension (SME) [34]. This carefully constructed model adds CPT-odd and Lorentz-violating contributions to the standard model; the shape of the considered corrections is inspired by effective field theory approaches applied to string theory. A comprehensive paper by Ding & Kostelecký [35] describes the derivation of SME coefficients based on experiments located at different coordinates and different alignment with respect to the rotational axis of the Earth's laboratory frame. In Penning traps, the SME interactions modify the energy levels of the trapped particle, which results in a CPT-odd frequency difference for quantum transitions involving a spin flip. By combining the BASE-Mainz data [14] with the BASE-CERN data [18] we are able to constrain energy difference modifications of the quantum-level structure resulting in limits on six combinations of SME coefficients. Table 1 summarizes previous upper bounds together with the updated numbers published in [18]. The recent BASE magnetic moment measurement improves the upper limits of the leading coefficient by a factor of 10–20.

**Table 1.** SME coefficients which are constrained by the BASE-Mainz and the BASE-CERN experiment. Second column: previous constraints as published in [35]. Third column: updated constraints published in [21].

coefficient	2016 constraint	2017 constraint
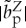	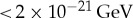	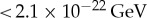
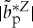	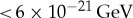	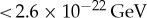
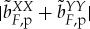	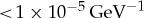	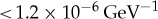
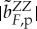	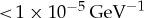	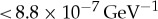
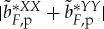	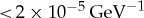	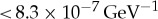
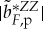	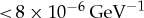	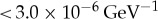

## 5. BASE physics in the ELENA era

The major goal of BASE in the ELENA era is the further improvement of measurement precision in 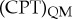 and 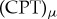.

The next logical step to improve 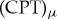 is the application of the double Penning trap technique to measure the magnetic moment of the antiproton. Major steps towards this goal have been achieved [19,21]. We anticipate that in first measurements after successful implementation of the double-trap method fractional precisions on the ppb level will be reached, with the potential to be further improved by at least a factor of 10. Highly stabilized superconducting trap magnets together with elegant phase-sensitive detection techniques [36] applied to measure the proton/antiproton oscillation frequencies will allow further reduction of the resonance line-widths.

The time budget of current magnetic moment measurements is limited by selective resistive cooling which is necessary to achieve single spin-flip resolution [14,19,22,24]. The implementation of sympathetic cooling of antiprotons by coupling the particles to laser-cooled Be^+^ ions using a common endcap method [37] or by direct Coulomb coupling in a micro-fabricated Penning trap [38], which are currently being prepared by collaboration members of BASE [37,38], will enable measurements at improved data-sampling rate. We anticipate that by combining all these techniques magnetic moment measurements at the 10 ppt level will become possible, in the long term.

Our 2014 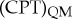 figure has reached limits imposed by the accelerator; another systematic error arose from systematic frequency shifts caused by residual inhomogeneities of the traps magnetic field [13]. To further improve 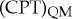 we have developed a self-shielding solenoid system with an improved shielding factor, reduced the magnetic field inhomogeneity of the trap and implemented tuneable axial detection systems which enable measurements in a static electric field [39] to further reduce systematic limitations. By combining these technical upgrades, we expect to improve the current best proton-to-antiproton charge-to-mass ratio comparison by at least a factor of 5. In addition, it is planned to implement the Pritchard two-particle phase method [40]. This technique makes use of simultaneous cyclotron frequency measurements using co-trapped particles on phase-locked magnetron orbits and is insensitive to first-order external magnetic field fluctuations.

## 6. Conclusion

Since the approval in 2013, the BASE collaboration has carried out the most precise comparison of the proton-to-antiproton charge-to-mass ratio [13], the most precise measurements of the proton [14] and antiproton magnetic moments [18], and has set the most stringent limit on directly measured antiproton lifetime [17]. Very recently, we resolved single spin flips with a single trapped antiproton [19] which is a major step towards an at least 100-fold improved measurement of 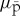. We anticipate that in the ELENA era the uncertainty in both numbers 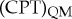 and 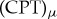 can be improved to the level of 10 ppt and 1 ppt, respectively.

## Data accessibility

All data presented in this paper will be provided on reasonable request.

## References

[1] Lueders G 1957 Proof of the TCP theorem. Ann. Phys. 2, 1 (10.1016/0003-4916(57)90032-5)

[2] Schwingenheuer B et al. 1995 CPT tests in the neutral kaon system. Phys. Rev. Lett. 74, 4376–4379. (10.1103/PhysRevLett.74.4376) 10058491

[3] Van Dyck RS, Schwinberg PB, Dehmelt HG 1987 New high-precision comparison of electron and positron *g*-factors. Phys. Rev. Lett. 59, 26–29. (10.1103/PhysRevLett.59.26) 10035093

[4] Bennett GW et al. 2006 Final report of the E821 muon anomalous magnetic moment measurement at BNL. Phys. Rev. D 73, 072003 (10.1103/PhysRevD.73.072003)

[5] Ahmadi M et al. 2017 Observation of the 1S/2S transition in trapped antihydrogen. Nature 541, 506–510. (10.1038/nature21040) 28005057

[6] Gabrielse G et al. 2012 Trapped antihydrogen in its ground state. Phys. Rev. Lett. 108, 113002 (10.1103/PhysRevLett.108.113002) 22540471

[7] Kuroda N et al. 2014 A source of antihydrogen atoms for in flight hyperfine spectroscopy. Nat. Commun. 5, 3089–3092. (10.1038/ncomms4089) 24448273 PMC3945878

[8] Ahmadi M et al. 2017 Observation of the hyperfine spectrum of antihydrogen. Nature 548, 66 (10.1038/nature23446) 28770838

[9] Hori M et al. 2016 Buffer-gas cooling of antiprotonic helium to 1.5 to 1.7 K, and antiproton-to-electron mass ratio. Science 354, 610 (10.1126/science.aaf6702) 27811273

[10] Gabrielse G, Khabbaz A, Hall D, Heimann C, Kalinowsky H, Jhe W 1999 Precision mass spectroscopy of the antiproton and proton using simultaneously trapped particles. Phys. Rev. Lett. 82, 3198–3201. (10.1103/PhysRevLett.82.3198)

[11] DiSciacca J et al. 2013 One-particle measurement of the antiproton magnetic moment. Phys. Rev. Lett. 110, 130801 (10.1103/PhysRevLett.110.130801) 23581304

[12] Smorra C et al. 2015 The BASE experiment. Eur. Phys. J. Spec. Top. 224, 3055 (10.1140/epjst/e2015-02607-4)

[13] Ulmer S et al. 2015 High-precision comparison of the antiproton-to-proton charge-to-mass ratio. Nature 524, 196 (10.1038/nature14861) 26268189

[14] Mooser A et al. 2014 Direct high-precision measurement of the magnetic moment of the proton. Nature 509, 596 (10.1038/nature13388) 24870545

[15] Ulmer S (2012).

[16] Smorra C et al. 2015 A reservoir trap for antiprotons. Int. J. Mass Spectrom. 389, 10 (10.1016/j.ijms.2015.08.007)

[17] Sellner S et al. 2017 Improved limit on the directly measured antiproton lifetime. New J. Phys. 19, 083023 (10.1088/1367-2630/aa7e73)

[18] Nagahama H et al. 2017 Sixfold improved single particle measurement of the antiproton magnetic moment. Nat. Commun. 8, 14084 (10.1038/ncomms14084) 28098156 PMC5253646

[19] Smorra C et al. 2017 Observation of individual spin quantum transitions of a single antiproton. Phys. Lett. B 769, 1 (10.1016/j.physletb.2017.03.024)

[20] Brown LS, Gabrielse G 1986 Geonium theory: physics of a single electron or ion in a Penning trap. Rev. Mod. Phys. 58, 233–311. (10.1103/RevModPhys.58.233)

[21] Nagahama H et al. 2016 Highly sensitive superconducting circuits at ∼700 kHz with tunable quality factors for image-current detection of single trapped antiprotons. Rev. Sci. Instrum. 86, 113305 (10.1063/1.4967493) 27910537

[22] Ulmer S, Rodegheri CC, Blaum K, Kracke H, Mooser A, Quint W, Walz J 2011 Observation of spin flips with a single trapped proton. Phys. Rev. Lett. 106, 253001 (10.1103/PhysRevLett.106.253001) 21770638

[23] Brown LS 1985 Geonium lineshape. Ann. Phys. 159, 62–98. (10.1016/0003-4916(85)90192-7)

[24] Mooser A et al. 2013 Resolution of single spin flips of a single proton. Phys. Rev. Lett. 110, 140405 (10.1103/PhysRevLett.110.140405) 25166966

[25] Häffner H et al. 2003 Double Penning trap technique for precise *g* factor determinations in highly charged ions. Eur. Phys. J. D 22, 163–182. (10.1140/epjd/e2003-00012-2)

[26] Geer SH et al. 2000 New limit on CPT violation. Phys. Rev. Lett. 84, 590 (10.1103/PhysRevLett.84.590) 11017323

[27] Geer SH, Kennedy DC 2000 A new limit on the antiproton lifetime. Astrophys. J. 532, 648 (10.1086/308552)

[28] Kobayashi K et al. 2005 Search for nucleon decay via modes favored by supersymmetric grand unification models in Super-Kamiokande-I. Phys. Rev. D 72, 052007 (10.1103/PhysRevD.72.052007)

[29] Babu KS, Pati JC, Wilczek F 1998 Suggested new modes in supersymmetric proton decay. Phys. Lett. B 423, 337 (10.1016/S0370-2693(98)00108-7)

[30] Hughes RJ, Holzscheiter MH 1991 Constraints on the gravitational properties of antiprotons and positrons from cyclotron-frequency measurements. Phys. Rev. Lett. 66, 854 (10.1103/PhysRevLett.66.854) 10043923

[31] Mooser A et al. 2013 Demonstration of the double Penning-trap technique with a single proton. Phys. Lett. B 723, 78–81. (10.1016/j.physletb.2013.05.012)

[32] Winkler PF, Kleppner D, Myint T, Walther FG 1972 Magnetic moment of the proton in bohr magnetons. Phys. Rev. A 5, 83 (10.1103/PhysRevA.5.83)

[33] Stadnik YV, Roberts BM, Flambaum VV 2014 Tests of CPT and Lorentz symmetry from muon anomalous magnetic dipole moment. Phys. Rev. D 90, 045035 (10.1103/PhysRevD.90.045035)

[34] Kostelecký VA, Russell N 2011 Data table for Lorentz and CPT violation. Rev. Mod. Phys. 83, 11 (10.1103/RevModPhys.83.11)

[35] Ding Y, Kostelecký VA 2016 Lorentz-violating spinor electrodynamics and Penning traps. Phys. Rev. D 94, 056008 (10.1103/PhysRevD.94.056008)

[36] Sturm S, Wagner A, Schabinger B, Blaum K 2011 Phase-sensitive cyclotron frequency measurements at ultralow energies. Phys. Rev. Lett. 107, 143003 (10.1103/PhysRevLett.107.143003) 22107189

[37] Bohman M (2017). https://arxiv.org/abs/1709.00433.

[38] Niemann M, Paschke A-G, Dubielzig T, Ulmer S, Ospelkaus C. 2014 CPT test with (anti)- proton magnetic moments based on quantum logic cooling and readout. In CPT and Lorentz symmetry: proceedings of the sixth meeting, Bloomington, IN, 17–21 June 2013 (ed. Kostelecký VA), pp. 41–44. Singapore: World Scientific (10.1142/9789814566438_0011)

[39] Heise F et al. 2017 High-precision measurement of the proton's atomic mass. Phys. Rev. Lett. 119, 033001 (10.1103/PhysRevLett.119.033001) 28777624

[40] Rainville S et al. 2004 An ion balance for ultra-high-precision atomic mass measurements. Science 303, 334 (10.1126/science.1092320) 14671311

